# Chronic pain, spasticity, and maternal burnout in children with spastic cerebral palsy

**DOI:** 10.1590/1806-9282.20252012

**Published:** 2026-06-29

**Authors:** Özge Baykan Çopuroğlu, Müge Baykan, Ahmet Özdemir

**Affiliations:** 1 Kayseri University, Department of Therapy and Rehabilitation – Kayseri, Türkiye.; 2 Recep Tayyip Erdoğan University, Rize Training and Research Hospital, Department of Pediatric Neurology – Rize, Türkiye.; 3 University of Health Sciences, Kayseri City Hospital, Department of Neonatology – Kayseri, Turkey.

**Keywords:** Cerebral palsy, Chronic pain, Spasticity, Burnout, Caregiver burden

## Abstract

**OBJECTIVE:**

The aim of this study was to examine the relationship between chronic pain, spasticity, and maternal burnout in children with spastic cerebral palsy, and to determine whether pain and muscle tone independently predict caregiver exhaustion.

**METHODS:**

This cross-sectional study included 126 mother–child dyads with spastic cerebral palsy aged 6–18 years. Pain was assessed using the Faces Pain Scale-Revised and revised Face, Legs, Activity, Cry, Consolability; spasticity using the Modified Ashworth Scale; and functional status by Gross Motor Function Classification System. Maternal burnout was evaluated with the Parental Burnout Assessment, and anxiety/depression with the Hospital Anxiety and Depression Scale.

**RESULTS:**

Mean pain score was 4.1±1.7, Modified Ashworth Scale 2.6±0.8, and maternal Parental Burnout Assessment 56.7±13.9. Burnout correlated significantly with pain (r=0.52), spasticity (r=0.44), Gross Motor Function Classification System level (r=0.39), caregiving hours, and Hospital Anxiety and Depression Scale-depression (all p<0.001). In regression analysis, pain (β=0.37), maternal depression (β=0.31), spasticity (β=0.24), Gross Motor Function Classification System level, caregiving time, and epilepsy were independent predictors of burnout (adjusted R^2^=0.56).

**CONCLUSION:**

Pain and spasticity are key contributors to maternal burnout in spastic cerebral palsy. Integrating pain and tone management into family-centered care may improve both child outcomes and caregiver well-being.

## INTRODUCTION

Children with spastic forms of cerebral palsy (CP) represent a population with complex motor, musculoskeletal, and psychosocial challenges. Spasticity and chronic pain are prevalent comorbidities in this group, substantially influencing functional ability, quality of life, and participation in daily activities^1^. Recent studies suggest that chronic pain in children with CP is under-recognized and often inadequately managed, despite its association with increased muscle tone, musculoskeletal deformities, and impaired mobility^2^. Moreover, the burden of caring for a child with spastic CP extends beyond the child: parents, and in particular mothers, often experience high levels of physical, emotional, and social strain. The concept of maternal burnout—a state of exhaustion, detachment, and reduced efficacy in the parenting role—has been increasingly explored in the context of chronic care and disability^3^. Studies have shown that parents of children with CP report higher burnout, which is correlated with the severity of the child's disability, ambulation status, and associated comorbidities^4,5^. Despite these individual lines of research, the interrelationships among child factors (chronic pain and spasticity/muscle tone) and maternal outcomes (burnout) remain insufficiently addressed^6^. Understanding how children's chronic pain and increased muscle tone may contribute to maternal burnout could inform holistic family-centered interventions in rehabilitation and pediatric neurology. Therefore, the present study aims to examine the associations among chronic pain intensity, spasticity, and maternal burnout in a cohort of children with spastic CP and their mothers. In addition, this study aims to determine whether pain and spasticity independently contribute to maternal burnout beyond functional severity and clinical comorbidities, thereby clarifying their relative impact on caregiver-related psychological outcomes. Elucidating these relationships may help identify potential targets for intervention that not only improve child outcomes but also reduce caregiver burden, enhance family well-being, and optimize rehabilitation strategies. Furthermore, a better understanding of these relationships may contribute to the development of targeted clinical approaches, including individualized pain management strategies, spasticity-focused rehabilitation, and structured caregiver support interventions within a family-centered care model.

## METHODS

The data supporting this research were collected from participants at Kayseri City Hospital, Türkiye, following institutional ethical approval and informed consent procedures.

This cross-sectional study was conducted between October 2024 and October 2025 in the Pediatric Neurology outpatient clinics of a tertiary care hospital, in accordance with the ethical principles of the Declaration of Helsinki. Ethical approval was obtained from the Kayseri City Hospital Ethics Committee (Approval No: 22), and written informed consent was obtained from all participating mothers. Mother–child dyads were enrolled if the child had a confirmed diagnosis of spastic CP established by a pediatric neurologist, was aged 6–18 years, and classified within levels I–V of the Gross Motor Function Classification System (GMFCS). Mothers were included if they were the primary caregiver for at least six months and aged between 18 and 65 years. Exclusion criteria were non-spastic types of CP (dyskinetic, ataxic, or mixed), acute illness or hospitalization within the previous 4 weeks; orthopedic surgery or intrathecal baclofen/botulinum toxin injection in the past 3 months; progressive neuromuscular disease; or maternal diagnosis of major psychiatric disorder under treatment.

The required sample size was calculated using G*Power 3.1 software for multiple linear regression analysis. Assuming a moderate effect size of f^2^=0.15, α=0.05, statistical power (1–β)=0.80, and six predictors (pain severity, spasticity, GMFCS level, epilepsy, child age, and caregiving duration), the minimum required sample size was 97 participants. This was based on effect sizes reported in a similar study investigating pain, spasticity, and caregiver burden in children with spastic CP^7^. To compensate for potential dropouts or incomplete data (~20%), a target sample of 120 mother–child pairs was planned.

Data collection was performed during a single face-to-face clinical visit. Mothers first completed a sociodemographic form and standardized questionnaires in a quiet room. Subsequently, each child underwent a structured physical examination by a pediatric physiotherapist blinded to caregiver-reported questionnaires. Spasticity was assessed using the Modified Ashworth Scale (MAS) in the elbow flexors, wrist flexors, hip adductors, hamstrings, and gastrocnemius muscles; the highest score was recorded as the global spasticity level.

All MAS assessments were performed by the same experienced pediatric physiotherapist to ensure inter-rater consistency. Children were evaluated in a standardized supine position after a resting period of at least 10 min to minimize the influence of activity-related changes in muscle tone. Passive movements were applied at a consistent speed according to standard MAS procedures.

Gross motor functional severity was categorized according to GMFCS levels I–V. Pain was assessed using the Faces Pain Scale-Revised (FPS-R) in communicative children (>4 years) and the revised Face, Legs, Activity, Cry, Consolability (r-FLACC) scale in non-communicating children; three observations were averaged for reliability. Comorbidities such as epilepsy, anticonvulsant or antispastic drug use, sleep disturbances, and constipation were documented through maternal report and medical records.

Information regarding ongoing rehabilitation was also recorded, including whether the child was receiving regular physiotherapy and the frequency of weekly sessions, to better characterize clinical management factors that may influence caregiver burden.

Maternal burnout was measured using the Parental Burnout Assessment (PBA), validated in Turkish populations, which consists of 23 items rated on a seven-point Likert scale (0–6) across four domains: emotional exhaustion in the parental role, contrast with previous parental self, feelings of being overwhelmed, and emotional distancing^8^. Higher scores indicate greater burnout. Maternal depressive and anxiety symptoms were evaluated using the Hospital Anxiety and Depression Scale (HADS) and included as covariates. Although both HADS-Anxiety and HADS-Depression scores were collected, only HADS-Depression was included in the regression model due to its stronger conceptual and empirical association with burnout and to reduce potential multicollinearity between emotional distress variables.

Maternal age, education level, employment status, socioeconomic status, duration of caregiving (years), number of caregiving hours per day, and number of children were also recorded to adjust for confounding factors.

Data were analyzed using SPSS version 26.0 (IBM Corp., Armonk, NY, USA). The normality of continuous variables was assessed using the Shapiro-Wilk test, histograms, and Q–Q plots. Descriptive data were presented as mean±standard deviation (SD), median (interquartile range), or frequencies (percentages), as appropriate. Bivariate correlations between pain scores, MAS scores, GMFCS levels, and maternal burnout were analyzed using Pearson or Spearman tests according to distribution patterns. HADS-Depression was included in the correlation analysis given its relevance to burnout, whereas HADS-Anxiety was not included in further analyses to avoid redundancy and overlapping variance with depressive symptoms. A multiple linear regression model was constructed with maternal burnout (PBA total score) as the dependent variable and child pain intensity, MAS score, and GMFCS level as the main independent variables. Child age, presence of epilepsy, caregiving duration, and maternal HADS scores were included as covariates. Multicollinearity was examined using the variance inflation factor (VIF<5). Statistical significance was set at p<0.05 (two-tailed). Missing data (<5%) were observed primarily in questionnaire-based variables (PBA and HADS). Multiple imputation was applied using fully conditional specification, and the imputed dataset was used in all subsequent analyses.

## RESULTS

A total of 143 mother–child dyads were assessed for eligibility; 126 fulfilled the inclusion criteria and were analyzed. Seventeen dyads were excluded due to non-spastic CP (n=5), recent orthopedic surgery or botulinum toxin injection (n=3), or incomplete maternal questionnaires (n=9). No adverse events or missing data exceeding 5% were observed.

The mean age of the children was 14.2±3.1 years, and 57.1% were male. Nearly half of the sample was classified as GMFCS levels IV–V (49.2%). The mean spasticity score was 2.6±0.8, while the mean pain score using FPS-R or r-FLACC was 4.1±1.7. Epilepsy was present in 41.3% of the children. The mothers had a mean age of 35.8±6.2 years, with an average daily caregiving time of 9.3±4.1 h. The mean PBA total score was 56.7±13.9, indicating moderate to high burnout levels. Mean HADS-Anxiety and HADS-Depression scores were 9.2±3.8 and 8.6±4.1 (Table 1).

**Table 1:** Demographic and clinical characteristics of children and mothers (n=126).

Variable		Mean±SD or n (%)
Child characteristics		
	Age (years)	14.2±3.1
	Male sex	72 (57.1)
	GMFCS Level I	14 (11.1)
	GMFCS Level II	22 (17.5)
	GMFCS Level III	28 (22.2)
	GMFCS Level IV	31 (24.6)
	GMFCS Level V	31 (24.6)
	MAS score (0–4)	2.6±0.8
	Pain score (FPS-R and r-FLACC)	4.1±1.7
	Epilepsy	52 (41.3)
Maternal characteristics		
	Age (years)	35.8±6.2
	Daily caregiving duration (h)	9.3±4.1
	PBA total score	56.7±13.9
	HADS-anxiety	9.2±3.8
	HADS-depression	8.6±4.1

SD: standard deviation; GMFCS: Gross Motor Function Classification System; MAS: Modified Ashworth Scale; FPS-R: Faces Pain Scale-Revised; r-FLACC: revised Face, Legs, Activity, Cry, Consolability; PBA: Parental Burnout Assessment; HADS: Hospital Anxiety and Depression Scale.

Maternal burnout demonstrated significant positive correlations with child pain intensity (r=0.52, p<0.001), spasticity (r=0.44, p<0.001), GMFCS level (r=0.39, p<0.001), caregiving hours per day (r=0.35, p<0.001), presence of epilepsy (r=0.28, p=0.002), and maternal depressive symptoms (HADS-D) (r=0.48, p<0.001). HADS-Anxiety was not included in the correlation analysis in line with the predefined analytical approach focusing on depressive symptoms as the primary emotional determinant of burnout. No significant correlation was found between maternal burnout and child sex or maternal educational status (p>0.05) (Table 2).

**Table 2:** Correlations between child clinical variables and maternal burnout (Parental Burnout Assessment total score).

Variable	r	p
Pain score	0.52	<0.001
MAS score	0.44	<0.001
GMFCS level	0.39	<0.001
Epilepsy (yes/no)	0.28	0.002
Caregiving duration (h/day)	0.35	<0.001
HADS-depression	0.48	<0.001

MAS: Modified Ashworth Scale; GMFCS: Gross Motor Function Classification System; HADS: Hospital Anxiety and Depression Scale.

A multiple linear regression was conducted to determine the independent predictors of maternal burnout. The overall model was significant [F(6,119)=26.4, p<0.001], explaining 56% of the variance in burnout (adjusted R^2^=0.56). Pain score was the strongest predictor (β=0.37, p<0.001), followed by maternal depressive symptoms (β=0.31, p<0.001), spasticity level (β=0.24, p=0.004), caregiving hours (β=0.16, p=0.014), GMFCS level (β=0.18, p=0.026), and epilepsy (β=0.13, p=0.041). Multicollinearity was not detected (all VIF values<2.0) (Table 3).

**Table 3:** Multiple linear regression predicting maternal burnout (Parental Burnout Assessment total score).

Predictor	β (standardized)	95%CI	p
Pain score	0.37	0.19–0.52	<0.001
MAS score	0.24	0.07–0.38	0.004
GMFCS level	0.18	0.02–0.33	0.026
Epilepsy	0.13	0.01–0.25	0.041
Caregiving hours	0.16	0.06–0.31	0.014
HADS-depression	0.31	0.14–0.47	<0.001

Model statistics: F(6,119)=26.4, p<0.001; Adjusted R^2^=0.56; VIF<2.0 for all predictors. CI: confidence interval; MAS: Modified Ashworth Scale; GMFCS: Gross Motor Function Classification System; HADS: Hospital Anxiety and Depression Scale.

## DISCUSSION

This study investigated the associations between chronic pain, spasticity, and maternal burnout in children with spastic CP. Findings indicated that both pain intensity and spasticity were independently associated with increased maternal burnout, even after controlling for functional severity, presence of epilepsy, daily caregiving duration, and maternal depression. Pain was the strongest predictor, followed by maternal depressive symptoms and muscle tone. These results suggest that child-related neuromuscular symptoms contribute significantly to parental psychological burden and should not be evaluated solely as physical impairments.

The rate of chronic pain in this cohort corresponds with existing literature, which documents a 50–75% pain prevalence in children with CP, especially in those presenting with severe motor disability and spasticity^9-11^. Pain in CP often arises from muscle overactivity, joint contractures, and hip displacement and is frequently underdiagnosed or undertreated^12^. The observed strong correlation between higher pain scores and maternal burnout aligns with previous findings indicating that parental distress increases in parallel with a child's discomfort and behavioral suffering^13,14^. Previous research similarly reported that greater pain severity in children with CP was associated with higher caregiver strain and reduced maternal quality of life^15^.

Spasticity was also a significant predictor of maternal burnout. Spasticity contributes not only to movement limitation and contracture development but also increases caregiving difficulty, positioning challenges, and pain^16^. Mediation analysis indicates that a portion of the effect of spasticity on maternal burnout is indirectly mediated by pain, supporting the hypothesis that spasticity-related pain serves as a mechanistic pathway linking motor impairment to caregiver distress. This observation is supported by previous evidence showing that spasticity reduction after botulinum toxin improves sleep, ease of care, and caregiver well-being^17,18^.

Functional severity was mildly to moderately associated with maternal burnout, which aligns with earlier studies demonstrating that higher motor impairment increases caregiver burden, particularly in non-ambulatory children^19,20^. The presence of epilepsy emerged as a secondary yet significant predictor, similar to prior research indicating that uncontrolled seizures elevate parental anxiety and exhaustion^21^. Daily caregiving hours also contributed independently, suggesting that time-dependent physical fatigue coexists with emotional exhaustion in chronic caregiving.

This study provides important clinical implications. First, assessment of pain and spasticity should be integral not only to child rehabilitation but also to caregiver mental health screening. Second, family-centered care models should incorporate parental burnout assessment tools such as the PBA to identify at-risk families. Third, early application of pain management, spasticity treatment, and respite care services may help prevent chronic parental exhaustion and its consequences, including depression, detachment, and reduced caregiving quality.

In clinical practice, pain management strategies may include pharmacological approaches (e.g., analgesics and antispastic agents), physiotherapy-based interventions (such as stretching, positioning, and manual therapy), and multidisciplinary pain management programs. Similarly, spasticity management may involve individualized rehabilitation programs, botulinum toxin applications, orthotic support, and caregiver training in daily handling and positioning techniques. Structured caregiver education and psychosocial support programs, including counseling and respite care, may further reduce caregiver burden and improve overall family functioning. Strengths include a relatively large sample, use of validated scales (r-FLACC, MAS, and PBA), adjustment for multiple confounders, and motor severity classification by GMFCS.

However, some limitations must be acknowledged. The cross-sectional design prevents causal inferences. Pain was measured using observational and self-report scales rather than quantitative sensory testing. Maternal burnout was self-reported and may have been influenced by social desirability bias. The study included only biological mothers; results may not generalize to other caregivers. Additionally, although information on rehabilitation status was recorded, variations in physiotherapy frequency, intensity, and treatment content were not analyzed in detail, which may have influenced both pain levels and caregiver burden. Future studies should incorporate detailed rehabilitation parameters to better understand their moderating effects.

Future research should use longitudinal designs to explore whether pain reduction or spasticity interventions lead to measurable decreases in parental burnout. Additionally, neuroimmunological biomarkers might elucidate biological links between chronic stress and caregiving.

## CONCLUSION

Chronic pain and spasticity in children with spastic CP are not only clinical impairments affecting the child, but also significant determinants of maternal burnout. In this study, pain intensity emerged as the strongest predictor of parental exhaustion, followed by muscle tone, functional severity, caregiving duration, maternal depressive symptoms, and epilepsy. These findings emphasize that effective management of pain and spasticity should be considered not only a child-centered intervention but also a family-centered health priority. Integrating systematic pain assessment, individualized spasticity management, and caregiver-focused psychosocial support into routine clinical practice may provide a comprehensive approach to reducing caregiver burden. Routine assessment of parental burnout, along with targeted pain control, spasticity treatment, and psychosocial support, may reduce caregiver strain and enhance overall rehabilitation outcomes. Longitudinal studies are needed to confirm causality and to evaluate whether interventions targeting pain and motor impairment can alleviate burnout in caregivers.

Future research should also explore multidisciplinary and family-centered intervention models to better address the complex interaction between child-related clinical factors and caregiver well-being.

## Data Availability

The datasets generated and/or analyzed during the current study are available from the corresponding author upon reasonable request.
